# Teething*Read before the Medical Association of Georgia, April, 1894.

**Published:** 1894-05

**Authors:** H. McHatton

**Affiliations:** Macon, Ga.


### Southern Medical Record.

A MONTHLY JOURNAL OF MEDICINE AND SURGERY.

VOL. XXIV. ATLANTA, GA., MAY, 1894. No. 5.

### Original Articles.

### TEETHING.*

BY H. McHATTON, M.D., MACON, GA.

There are a class of popular diseases that one naturally attacks with a certain amount of hesitancy, as the task is so great that he fears entering into it. Among this class may be mentioned "Change of Life," Biliousness, synonym "My liver don't act," and last, but by no means least, "Teething." This group of diseases, in my estimation, cause more preventable deaths in our State than any other we can mention.

It does seem that in writing an article on "Teething" one would be safe in saying what he pleased, but think of what he directly antagonizes: Tradition, Superstition, Ignorance, the monthly nurse, the grandmother, every old woman in the neighborhood, married, single or otherwise, and last, but by no means least, the medical profession.

The doctors may be divided into three classes in their attitude toward this disease:

1st. Those that in all cases attempt to make a correct diagnosis of the condition of their patient, and go into a long and usually thankless course of explanation as to the supposed case of teething

*Read before the Medical Association of Georgia, April, 1894.

being due to dietary indiscretions, improper care, inherited defects, or any other condition that is so commonly diagnosed as teething.

2d. Those that know that the condition is due to some other cause, but who are of that type that avoid all worry or extra work, and will treat the case on its merits, but are perfectly willing to allow the family to worship their idol of teething to their heart's content.

3d. Those that foster and directly propagate the disease in the popular mind, some because the diagnosis of teething is always acceptable. No one is usually blamed for the death of a teething child, it is so simple, so easy, so satisfactory and so mutually agreeable to all parties in the case, with the probable exception of the baby, who fortunately has nothing to say. Others who unfortunately have no diagnostic ability, and still a few others who honestly and conscientiously believe that dentition is directly the cause of most of the ills of infantile life.

No one denies that dentition is a physiological process. *Pari passu* as the infant departs from its natural state, teething is increased in violence. The infant who is fortunate enough to have a mother who is physiologically perfect, and who treats her child as nature intended, does not teeth the first year, but is liable to the second summer, we all know why. The one who has to be partially bottle fed, teeths oftener and earlier than the above mentioned. The one that is bottle fed from the start, and exclusively, teeths, as a rule, early, often, and in many cases fatally. This is not confined to infants. All of us who have had much experience in raising the young of any of the mammals know that the mortality from teething bears the same proportion under the same circumstances as it does with the infant. Were the condition of teething due only to dentition, the surroundings of the child would not be so important a factor; neither would the season of the year. Yet it is well known that summer is pre-eminently the teething season.

In partial proof of the above, allow me to quote a few cases of so called teething. I have a patient who is the mother of ten children; she has always lived in the same place and in the same position of life. With the first eight children she had an abundance of milk and not a case of teething; with the last two she had no milk and two fatal cases of the disease. Another patient, mother of four children, same surroundings as the first, she never had any milk, first two children died teething; second two in consequence of employment of wet nurse have never had an attack, and are both past the period when it is possible. Another case, first child born in the fall, mother with an abundance of milk, but the idea that the good Lord did not know what an infant should be fed on. This infant began to have attacks of teething early in the spring, each one easily traced to some dietic spree, and each one more severe than the previous. At last a severe attack, following a meal of corn bread and pot-liquor, a rather plain talk on my part brought out the fact that doctors did not know much about teething children anyway, and no doctor had the experience Aunt Lucy had had. I had it, Aunt Lucy was my professional rival, and I felt the time had come when our respective positions should be defined. Upon being called, Aunt Lucy proved to be one of the regular old ante-bellum type of negro nurses, that cannot be bettered if they are controlled. We conversed as follows:

"Been nursing long, Auntie?"

"Yes, sir; nussin' young miss dare, an' ole miss an' all ole missus' chullun since afore freedom."

"Is pot-liquor and corn bread good for babies?"

"Corn bread and pot-liquor's de bes' in de world for babies an' puppies, yes, sir."

"Always feed your own babies on it?"

"Yes, sir."

"How many babies have you had, Aunty?"

"Thirteen, sir."

"How many alive now?"

"One, bless de Lawd."

"That will do, Aunty."

Upon the withdrawal of my rival I informed my patient that it was purely a question for her own decision, to try my plan of infantile feeding with an unknown mortality, or Aunt Lucy's with a mortality of twelve in thirteen. Since then I have had no trouble with teething children in that family.

Some years ago I saw in consultation a fatal case of teething in an infant of eight months. The mother was a perfect specimen of womanhood, with, as far as any test could reveal, an abundant and physiological secretion of milk. She assured me that there was no possible chance for the baby to have ever had anything but the breast milk, as her nurse was perfection, and the child was never from under her supervision. The case was an enigma to both the family physician and myself, why a child under these circumstances should die of a gastro-enteritis we could not see. Over a year afterwards, we found that for weeks previous to the fatal illness the nurse had taken the infant into the kitchen at each meal and fed it with everything on the table.

Last summer I had a very severe attack of gastro-enteritis in a fine infant of seven months, and never did succeed in convincing the family that it was not a case of teething or that the meal of grated ham, strawberries and ice-cream, taken the evening before the attack, could have had any bearing on the condition.

I saw an infant of three weeks being fed on coffee and light-bread, and know of another of three months in a public institution in this State that would eat nothing but greens and hominy ; both, of course, teethed promptly and fatally.

It is useless to multiply this type of cases to endeavor to show a relation between the so-called condition of teething and dietic errors. I suppose there is scarcely a disease of infancy that has not been diagnosed by the laity, and even the profession in times gone by, as due to teething, or being influenced by said condition. All such diagnostic errors lead to some fatal cases, but they all combined do not cause as many deaths as the diseases referable to the digestive system. It is only recently that a certain amount of looseness of the bowels in the teething infant has not been considered beneficial by the mass of the profession, on the same vague idea that one should not cure an ulcer of the lower limb, bleeding piles, fistula in ano, or an extensive eczema for fear of some grave consequences.

The looseness of the bowels and eczema in the teething infant you still occasionally hear of, the rest are things of the past, professionally. The looseness of the bowels fallacy flourishes and even extends from year to year among the laity, with our farmer friends. I may say that attacking it is on a par with attacking nut-grass, the more you fight it the worse it grows.

I know that most of us will agree that dietic errors are the cause of nearly all digestive disorders in the infant, and all of us, that whereas the said disorders are in the beginning and non-inflammatory stage most amenable to appropriate treatment, by neglect they become the worst type of diseases that we have to contend with. What would early in the case have been a question of a dose of oil and appropriate food, in a few days is a condition in which often our art avails us nothing, either in medicine, food or a radical change of climate. Right here is where the fatality of the popular idea of the beneficial effect of the loose bowels comes in.

How often have each of us been called in by the young mother, and the old one too, and gotten the following history: "Baby's bowels began to get loose a few days ago. She is teething, and you know that all teething babies have loose bowels. They are not getting any better and we thought we had better see you. She is having about a dozen actions a day, and throws up everything we give her." We are led to the little crib there is no use in describing what we see, we have all seen it, and so has nearly every mother in the land. We examine the little sufferer, try to look unconcerned, and at the same time say to ourselves, "too late." Let us, one and all, use our most earnest endeavors to correct as much as possible this popular and fatal fallacy.

I will now give some quotations from standard authors, and beg your indulgence for their length, as this is a subject of deep interest to me:

Forcheimer discourses in an exhaustive manner the symptoms and treatment of dentition. He believes dentition to be a physiological process, subject, perhaps, to occasional pathological conditions, but these are infrequent and obscure.

Brothers, after long study upon the subject, is convinced that the influence of teething has been much exaggerated, but he is not willing to join the ranks of those who deny it any place whatever. The teaching that moderate diarrhea during the period of dentition is beneficial is pernicious and has caused the death of many children.

Adams believes the evolution of the teeth to be a purely physiological process, subject sometimes to perversion. In a series of careful observations on 288 patients, he found 70 per cent. of those without teeth were fed wholly or in part upon table food. With such evidence the question at once arises, whether it is not more scientific to attribute digestive disturbance to a known factor, improper food, than to a hypothetical one, a tooth still confined to its sac.

Jacobi, in considering the morbid process which has been said to originate in normal dentition, says: "It should not be forgotten that dentition is a physiological process. I have not seen convulsions dependent upon difficult dentition in the course of the last ten years. A large number of diseases which have been attributed to dentition owe this erroneous diagnosis to the fact that the diagnostic powers of the practitioner were limited, like those of the public with which he had to deal."

Eustace Smith, speaking of chronic diarrhea: "In the case of infants who are cutting the incisors, these teeth usually appear without difficulty or apparent aggravation of other symptoms, nor does the eruption of each tooth appear to be accompanied by any improvement which can be attributed to that as its cause. Dentition goes on rapidly and easily, while the diarrhea remains stationary or slowly improves. Many children are said to cut their teeth with diarrhea; perhaps, however, dentition in these cases is not so entirely to be blamed as is commonly supposed."

J. Lewis Smith: "The opinion formerly entertained in the profession, and now prevalent in the community, that many infantile maladies arise, directly or indirectly, from dentition, is erroneous."

James Fenlayson, in his work on diagnosis in Keating's Cyclopedia, has a subdivision with this caption, "Diagnosis Made Easy —Teething." Some extracts are as follows: "This convenient theory of the dependence of infantile diseases on the process of dentition is of course now exploded, and it might be supposed that it needed no notice in a work like this, but superstitions are difficult to kill. A tooth rash is a splendid safety valve, and when it resists our best efforts at treatment, we can explain how dangerous it is to cure a rash in a teething child in case of its driving in the disease to some internal organ. The affection of the eyes and ears are too obvious to require explanation. Do we not speak of the eye-tooth, and who has not felt a pain in his ear from a bad molar?

"The popularity depends partly in its saving a world of trouble to the doctor, but also its meeting the views of the parents, but if the beginner is ever to make any progress in the diagnosis and treatment of the diseases of infancy, he must take up the attitude of refusing to believe that any child is ever seriously ill from teething."

John Downing: "At one time dentition was held accountable, directly or indirectly, for nearly all the ills of infancy. At the present day, owing to a more extended knowledge of the etiology of disease and greater proficiency in methods of diagnosis, the symptomatology of dentition and its power as an etiological factor are with the majority of the profession becoming more and more restricted, and by some totally ignored. There are, however, others who still adhere to the old doctrine of dentition as tenaciously as do the laity, and in their practice among the infant population find more use for the gum lancet than for common sense."

I have been in the habit for some time of giving a little pamphlet to each of my patients who have babies, and must say, after some years of trial, the schedule below has given more satisfaction than any method of infant feeding I have yet tried, and it has served as a most efficient prophylaxis against teething, where I can get it fully complied with:

### GENERAL RULES FOR FEEDING.

No artificial food can compare with mother's milk.

Diarrheal diseases are the greatest cause of mortality in young children.

Never neglect a diarrhea in a child because you think it is teething and the diarrhea is consequently natural.

| AGE. | INTERVALS OF FEEDING. | AVERAGE AMOUNT. AT EACH FEEDING. | AVERAGE AMOUNT IN 24 HOURS. |
|---|---|---|---|
| First Week. | 2 Hours. | 1 Ounce. | 10 Ounces. |
| One to Six Weeks. | 2 to 2½ Hours. | 1½ to 2 Ounces. | 12 to 16 Ounces. |
| Six to Twelve Weeks and Possibly to Fifth or Sixth Month. | 3 Hours. | 3 to 4 Ounces. | 18 to 24 Ounces. |
| At Six Months. | 3 Hours. | 6 Ounces. | 36 Ounces. |
| At Ten Months. | 3 Hours. | 8 Ounces. | 40 Ounces. |

Diet during the first week: *Cream, 2 teaspoonfuls; milk, 3 teaspoonfuls; water (hot), 3 teaspoonfuls; milk sugar, ¼ teaspoonful.

For each portion: To be given every two hours from 5 A. M. to 11 P. M., and in some cases once or twice at night.

Diet from the second to the sixth week: Milk, 1 tablespoonful; cream, 2 teaspoonfuls; milk sugar, ¼ teaspoonful; water, 2 tablespoonfuls.

For one portion: To be given every two hours from 5 A. M. to 11 P. M.

Diet from the sixth week to the end of the second month: Milk, 2½ tablespoonfuls; cream, 1 tablespoonful; milk sugar, ½ teaspoonful; water, 2½ tablespoonfuls.

For each portion: To be given every two hours.

Diet from the beginning of the third month to the sixth month: Milk, 5 tablespoonfuls; cream, 1 tablespoonful; milk sugar, 1 teaspoonful; water, 2 tablespoonfuls.

For each portion: To be given every two and a half or three hours.

Diet during the sixth month: Six meals daily from 6 or 7 A. M. to 9 or 10 P. M.

Morning and mid-day bottles each: Milk, 9 tablespoonfuls;

*Cream, milk and water to be sterilized.

cream, 1 tablespoonful; Mellin's Food, 1 teaspoonful; water (hot), 2 tablespoonfuls.

Dissolve the Mellin's Food in the hot water and add, with stirring, to the previously mixed milk and cream.

Other bottles each: Milk, 9 tablespoonfuls; cream, 1 tablespoonful; milk sugar, 1 teaspoonful; water, 2 tablespoonfuls.

In the seventh month the Mellin's Food may be increased to two teaspoonfuls and given three times daily.

Throughout the eighth and ninth months five meals a day will be sufficient.

First meal at 7 A. M.: Milk, 13 tablespoonfuls; cream, 1 tablespoonful; milk sugar, 1 teaspoonful; water, 2 tablespoonfuls.

Second meal at 10:30 A. M.: Milk, cream and water in the same proportions; Mellin's Food, one tablespoonful.

Third meal at 2 P. M.: Same as second.

Fourth meal at 6 P. M.: Same as second.

Fifth meal at 10 P. M.: Same as first.

Instead of Mellin's Food, a teaspoonful of "flour-ball" may be added.

Two meals of flour-ball daily—say the second and fourth—are all that can be digested. To prepare these, rub one teaspoonful of the powder with a tablespoonful of milk into a smooth paste, then add a second tablespoonful of milk, constantly rubbing until a cream-like mixture is obtained. This is poured into eight ounces of hot milk, stirring well, and is then ready for use. The other meals should be composed of milk, cream, sugar of milk and water, as already given.

Mellin's Food and flour-ball may be substituted by oatmeal or barley.

Diet for the the tenth and eleventh months: First meal 7 A. M.: Milk, 17 tablespoonfuls; cream, 1 tablespoonful; Mellin's Food, 1 tablespoonful.

Or, flour-ball or barley jelly, two teaspoonfuls; water (used only with Mellin's Food), two tablespoonfuls.

Second meal at 10:30 A. M.: A breakfast-cupful of warm milk (eight ounces).

Third meal at 2 P. M.: The yolk of an egg lightly boiled, with stale bread-crumbs.

Fourth meal at 6 P. M.: Same as first.

Fifth meal at 10 P. M.: Same as second.

On the alternate days the third meal may consist of a teacupful (six ounces) of beef tea containing a few stale bread-crumbs.

Diet from the twelfth to the eighteenth month: Five meals per day.

First meal at 7 A. M.: A slice of stale bread, broken and soaked in a breakfast-cupful (eight ounces) of milk.

Second meal at 10 A. M.: A teacupful of milk (six ounces) with a soda biscuit or thin slice of buttered bread.

Third meal at 2 P. M.: A teacupful of beef tea (six ounces) with a slice of bread, one good tablespoonful of rice and milk pudding.

Fourth meal at 6 P. M.: Same as first.

Fifth meal at 10 P. M.: One teaspoonful of Mellin's Food with a breakfast-cupful of milk.

To alternate with this:

First meal at 7 A. M.: The yolk of an egg lightly boiled, with bread-crumbs; a teacupful of milk.

Second meal at 10 A. M.: A teacupful of milk with a thin slice of buttered bread.

Third meal at 2 P. M.: A mashed boiled potato, moistened with four tablespoonfuls of beef tea; two good tablespoonfuls of junket.

Fourth meal at 6 P. M.: A breakfast-cupful of milk with a slice of bread broken up and soaked in it.

Fifth meal at 10 P. M.: Same as second.

The fifth meal is often unnecessary and sleep should never be disturbed for it; at the same time, should the child awake an hour or more before the first meal, he must break his fast upon a cup of warm milk, and not be allowed to go hungry until the set breakfast hour.

Diet from eighteen months to the end of two and one-half years, four meals a day:

First meal at 7 A. M.: A breakfast-cupful of milk; the yolk of an egg lightly boiled; two thin slices of bread and butter.

Second meal at 11 A. M.: A teacupful of milk with a soda biscuit.

Third meal at 2 P. M.: A breakfast-cupful of beef tea, mutton or chicken broth; a thin slice of stale bread; a saucer of rice and milk pudding.

Fourth meal at 6:30 P. M.: A breakfast-cupful of milk with bread and butter.

On alternate days:

First meal at 7 A. M.: Two tablespoonfuls of thoroughly cooked oat meal or wheaten grits with sugar and cream; a teacupful of milk.

Second meal at 11 A. M.: A teacupful of milk with bread and butter.

Third meal at 2 P. M.: One tablespoonful of under-done mutton pounded to a paste; bread and butter, or mashed baked potato moistened with good, plain dish gravy; a saucer of junket.

Fourth meal at 6:30 P. M.: A breakfast-cupful of milk, a slice of soft milk toast, or a slice or two of bread and butter.

When sickness supervenes, all that is ordinarily necessary is a reduction of the diet to plain milk, or milk with Mellin's Food.

Even the youngest infant requires water several times daily, and the demand increases with age.

The foregoing schedule must, of course, be regarded only as an average.

Many children can bear nothing but milk food up to the age of two, or even three years, and, provided enough be taken, no fear for their nutrition need be entertained. If a child be thriving on milk, he is never to be forced to take additional food merely because a certain age has been reached; let the healthy appetite be the guide.

OAT-MEAL GRUEL.—Mix a tablespoonful of oat meal with two tablespoonfuls of cold water, stirring to bring to a state of uniformity; then pour it into a pint of boiling water in a saucepan, and boil and stir well for ten minutes. Flavor with salt or sugar. If the boiling be continued for half an hour the mixture thickens into a porridge.

BARLEY WATER.—Put two teaspoon fuls of Robinson's Prepared Barley into a saucepan with a pint of clear water, and boil slowly down to two-thirds of a pint; strain through muslin.

FLOUR BALL.—Take a pound of good wheat flour, tie it up very tightly in a strong pudding bag; place it into a saucepanful of water and boil constantly for ten hours. When cold remove the cloth, cut away the soft outer covering of dough that has been formed. and reduce the hard, baked interior by grating.

WHEY.—Milk, one pint; essence of pepsin (Fairchild's) two teaspoonfuls. Heat the milk up to a point that can be agreeably borne by the mouth, and add the pepsin with gentle stirring; let the whole stand until firm coagulation has taken place, then beat with a fork until the curd is finely divided, and strain.

